# Binary Addition in Resistance Switching Memory Array by Sensing Majority

**DOI:** 10.3390/mi11050496

**Published:** 2020-05-14

**Authors:** John Reuben

**Affiliations:** Chair of Computer Science 3—Computer Architecture, Friedrich-Alexander-Universität Erlangen-Nürnberg (FAU), 91058 Erlangen, Germany; johnreuben.prabahar@fau.de

**Keywords:** memristor, resistance switching memory, non-volatile memory (NVM), in-memory computing, majority logic, adder, Sense Amplifier, Boolean logic

## Abstract

The flow of data between processing and memory units in contemporary computing systems is their main performance and energy-efficiency bottleneck, often referred to as the ‘von Neumann bottleneck’ or ‘memory wall’. Emerging resistance switching memories (memristors) show promising signs to overcome the ‘memory wall’ by enabling computation in the memory array. Majority logic is a type of Boolean logic, and in many nanotechnologies, it has been found to be an efficient logic primitive. In this paper, a technique is proposed to implement a majority gate in a memory array. The majority gate is realised in an energy-efficient manner as a memory READ operation. The proposed logic family disintegrates arithmetic operations to majority and NOT operations which are implemented as memory READ and WRITE operations. A 1-bit full adder can be implemented in 6 steps (memory cycles) in a 1T–1R array, which is faster than IMPLY, NAND, NOR and other similar logic primitives.

## 1. Introduction

**Figure 1:**
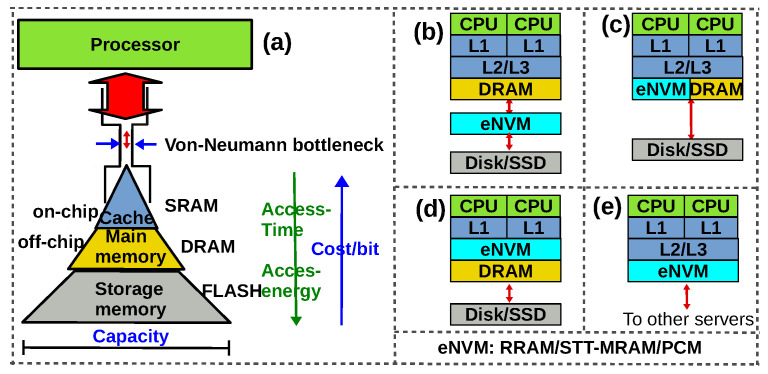
(**a**) The von-Neumann bottleneck. Possible ways in which emerging non-volatile memories (NVMs) (eNVM) can be integrated into the memory hierarchy (**b**) eNVM can fill the gap between Solid State Drive (SSD) and main memory (**c**) eNVMs can be integrated alongside main memory (**d**) eNVMs can be integrated as embedded memory (Last level cache replacement) (**e**) Universal memory (Main and storage) [1].

Contemporary computing systems exhibit a deep memory hierarchy. The reason for such a hierarchy is that the non-volatile memory technology (FLASH) used as storage memory has an access time of hundreds of microseconds, while processors clocked at GHz need to access data in nanoseconds (Figure 1). So small SRAMs that have sub-nanosecond access time are used as caches (which are in turn organised as Levels L1,L2,L3 with each level becoming larger and hence slower). If the data the processor is looking for is not in the cache L1, it looks for it in the next levels (L2 and L3), and then in main memory (DRAM) and so on [1]. Thus, the performance of computing systems (speed) is constrained by the availability of data and the farther it is from the processor, the more the time and energy to procure it. This is the ‘memory wall’: the mismatch in the performance (speed) of processor and memory, and the energy for memory access, which is growing exponentially along the memory hierarchy (from cache to main memory to storage memory, Figure 1). This ‘memory wall’, often referred to as the ‘von Neumann bottleneck’ is the main reason for degraded performance (and energy consumption) of present day computing systems. There has been an ongoing effort (since 10–15 years) to overcome the memory wall by bringing the processor and memory unit closer to each other. Early researchers used the term processing-in-memory (PIM) to refer to the effort to move the processing closer to where data resides. The term processing-in-memory broadly referred to processing in the memory using computing units placed in the memory chip. Later researchers used the term near-memory computing or near data processing to refer to the same effort and they exploited 3D stacking of DRAM dies over logic die to compute near memory. However, all these efforts try to minimize the physical distance between memory (where data is stored) and processing units and they do not completely solve the von-Neumann bottleneck because the need for data transfer persists.

Resistive-switching memories are a class of emerging Non-Volatile Memories (eNVMs) which store data as resistance. When subject to a voltage/current stress, the resistance can be changed between a Low Resistance State (LRS) and a High Resistance State (HRS) (hence the name resistance-switching). The word ‘memristor’ is also used by researchers to refer to a resistive-switching device since such a device is basically a ‘resistor’ with a ‘memory’. From the perspective of device physics, a memristor can be classified on the basis of its switching mechanisms as follows:

1. Resistive Random Access Memory (RRAM) device is a Metal-Insulator-Metal structure where a conductive filament is created (LRS) or broken (HRS) in the insulator. The insulator is usually a transition metal oxide (OxRAM) or an electrolyte (Conductive Bridge RAM)
2. Phase Change Memory (PCM) device is a Metal-Active Material-Metal structure where the active material is a chalcogenide phase-change material which is either in amorphous (HRS) or crystalline state (LRS)
3. Spin Transfer Torque-Magnetic RAM (STT-MRAM) is a Free layer-Tunnel Layer-Reference layer structure where the magnetic polarization of the Reference layer is fixed while that of the free layer can be programmed to be either in the same direction (parallel, LRS) or opposite direction (anti-parallel, HRS)

Currently, there is no consensus regarding the way in which such eNVMs can be integrated in the memory hierarchy. Four possibilities are envisioned, as shown in Figure 1b–e [1]. More details on the integration of each of the aforementioned memories into the memory hierarchy can be found in Reference [2]. To construct a memory array using such devices, two configurations are common: 1Transistor-1 Resistor (1T–1R) and 1Selector-1 Resistor (1S–1R). The 1T–1R configuration uses a transistor as an access device for each memory cell, allowing one to access a particular cell without interfering with its neighbours in the array. The 1S–1R configuration uses a two-terminal device called a ‘selector’ which has a diode-like characteristic. The selector is assembled in series with the memristive device. The 1S–1R is area-efficient, but suffers from sneak–path problem because it is not possible to program (read or write to a cell) a cell without interfering with its neighbours [3].

At a time when computer architects are facing the memory wall, the emergence of resistance switching devices has set the stage for them to be efficiently deployed for in-memory computing. The term ‘in-memory computing’ (also called ‘processing-in-memory’/‘compute-in-memory’) is used to refer to any effort to process data at the residence of data (i.e., in the memory array) without moving them to a separate processing unit. If one can compute at the residence of data, in principle, the memory wall problem could be solved since the demarcation (between processing unit and memory) and the accompanying costly data transfer can be eliminated. The word ‘processing/compute’ can mean a wide variety of tasks from computer arithmetic (binary addition/subtraction) to cognitive tasks like machine learning and pattern recognition. In this work, the focus is on computer arithmetic.

**Figure 2:**
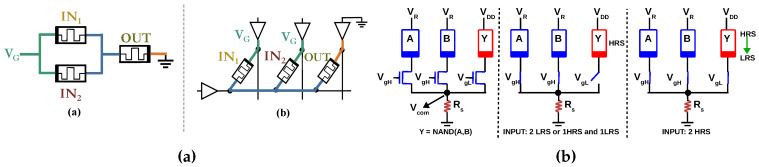
(**a**) Example of a NOR-based memristive logic family [6] (NOR gate (**a**) can be implemented by exploiting the fact that resistances $IN_{1}$ and $IN_{2}$ will be in parallel in the memory array (**b**). OUT which is initialized to Low Resistance State (LRS) will either switch to High Resistance State (HRS) or remain in the same state, depending on inputs $IN_{1}$ and $IN_{2}$ of the NOR gate which are stored as resistance of the memristor); (**b**) Example of a NAND-based memristive logic family [7] (A large voltage $V_{gH}$ is applied to the gates of A and B to guarantee that transistors are turned ON, while a relatively small voltage $V_{gL}$ is applied to the gate of output Y. $V_{DD}$ larger than $V_{R}$. $V_{com}$ is ≈0 when both inputs are HRS, while it charges to ≈0.8 V for (HRS,LRS) and (LRS,LRS) case, cutting off the access transistor of Y. This ensures the switching of Y only when both inputs are HRS [7]).

Conventionally, digital circuits have been implemented using logic gates built from CMOS transistors. In contrast, a memristive logic family formulates a ‘functionally complete’ Boolean logic using a memristive device (RRAM/PCM/STT-MRAM) as the primary switching device (CMOS circuitry may also be used, but in a peripheral manner). For example, NOR is ‘functionally complete’ since any Boolean logic can be expressed in terms of NOR gates. Therefore, if a NOR gate can be designed using memristive devices, any Boolean logic can be implemented using memristive devices. Furthermore, most researchers try to make their logic gates executable in an array configuration so that they can be exploited for in-memory computing. Other than NOR, NAND, IMPLY+FALSE [4] and Majority+NOT [5] are also functionally complete. As an example, the structure of a NOR-based memristive logic family in 1S–1R and a NAND-based memristive logic family in 1T–1R are shown in Figure 2. In this work, a Majority+NOT based memristive logic family is proposed.

**Figure 3:**
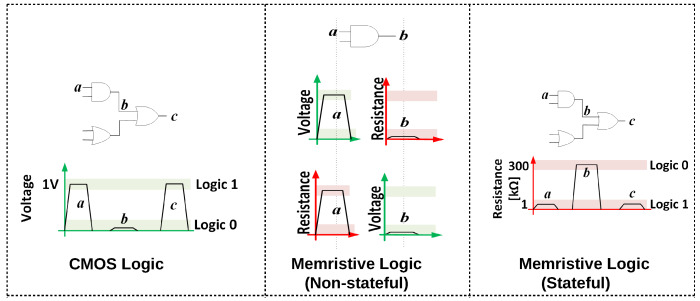
In CMOS logic, inputs, outputs and intermediate values are represented as voltages; in memristive logic, inputs, outputs and intermediate values are represented as either voltages or resistances. A memristive logic is said to be stateful if resistance is the only state variable for representing inputs, outputs and intermediate results of computation.

Many such logic families using basic Boolean gates (OR, NOR, AND, NAND, XOR) have been proposed in recent years. Thus memristive logic families can be classified based on their logic primitives as being NOR-based, NAND-based, majority-based and so forth. From another perspective, memristive logic differs fundamentally from conventional CMOS logic. In CMOS logic, there is only a single logic state variable, i.e., voltage. The input data is represented as voltage and is processed as voltage throughout the computation (including in all of the intermediate stages), and is finally also represented as voltage at the output, as illustrated in Figure 3. Furthermore, the state variable is regenerated throughout the computation by the CMOS gates. This seamless flow is disrupted in memristive logic because the internal state of memristors governs their resistance, introducing resistance, in addition to voltage, as a logic state variable for computation [8]. If resistance is the only state variable during the entire computation, a memristive logic family is said to be stateful. In certain logic families, the input is a voltage, but the output is a resistance [9] and in certain other families, the input is the resistance and the output of computation is a voltage [10]. These are classified as non-stateful logic families (Figure 3). Other than this overview of memristive logic families, we do not delve into the details of all the families. A detailed survey of all such works is beyond the scope of the paper and the reader is referred to surveys of such in-memory computing approaches elaborated in References [8,11,12].

Majority gate is a Boolean logic gate whose output is defined to be true if more than half of the *n* inputs are true, where *n* is odd. Majority logic is a logic primitive with expressive power, i.e., arithmetic operations can be expressed with less gates in majority logic, when compared to Boolean NAND/NOR [5]. In this paper, a ‘majority+NOT’ based memristive logic family is proposed. The proposed logic family computes majority by sensing the data stored in a 1T–1R array, i.e., the inputs of the majority gate are the resistances of the memristors and the output is sensed as a voltage. Therefore the proposed family is a non-stateful logic family. The remainder of the paper is organized as follows. Section 2.1 presents the principle of computing majority in a 1T–1R array. Since majority is ‘sensed’ in the proposed logic family, the Sense Amplifier (SA) is the crucial aspect of the proposed gate. Hence, we present the detailed sensing methodology in Section 2.2. Further, we investigate the amount of tolerance to variations in resistive states by performing simulations. We also generalize the approach to other RRAMs in Section 2.3. Section 3.1 briefly presents the design of the multi-row decoder needed for the proposed logic family. As an example, a 1-bit full-adder is implemented in a 1T–1R array using the proposed method (Section 3.2) followed by comparison with other in-memory adders (Section 3.3) and conclusion (Section 4).

## 2. Majority Logic in 1T–1R Array

### 2.1. Majority Gate: Principle of Operation and Validation

**Figure 4:**
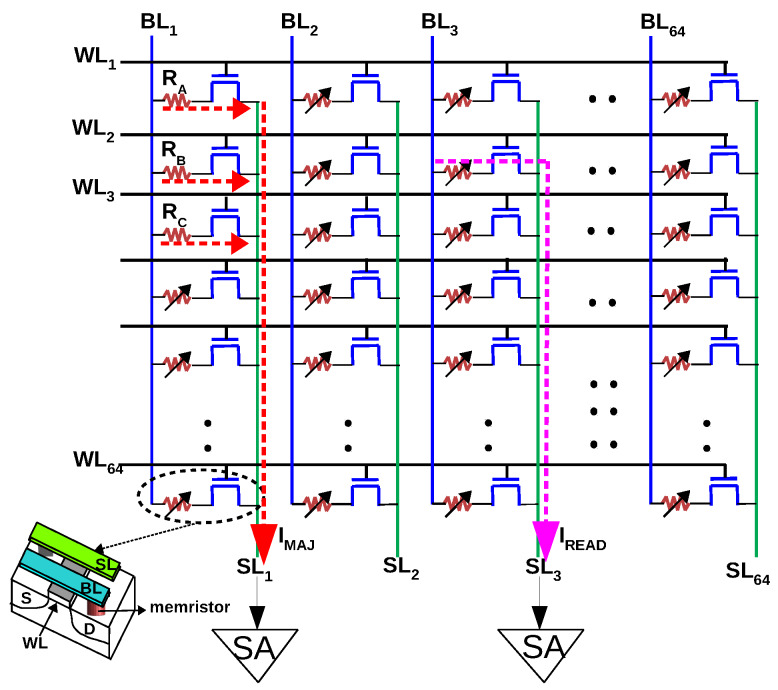
In a conventional memory, a single row is activated and $V_{R}$ is applied across the cell to read from it (e.g., reading from cell at (2,3) depicted in pink). To compute majority, three Word Lines ($WL_{1,2,3}$) are activated simultaneously and $V_{R}$/3 is applied across cells in column 1 resulting in $I_{MAJ}$.

The 1T–1R configuration uses a transistor as an ideal selector to isolate the accessed cell from its neighbours in the array. Although research in two terminal selectors (which can be integrated in series with RRAM) is active to solve the sneak-path problem, the 1T–1R configuration is a viable alternative to 1S–1R, as demonstrated by successful prototypes in both academia and industry. The absence of sneak currents makes writing and reading energy-efficient and error-free. The RRAM is SET/RESET by applying two pulses simultaneously to the WordLine (WL) and BitLine (BL)/SourceLine(SL). To read from a cell, the corresponding WL is activated (to switch ON the transistor) and a small voltage, $V_{R}$ is applied across the cell (BL-SL) and the current from the cell is collected at the SL and measured using a SA (Figure 4). We shall assume that the three Boolean inputs *A*, *B*, *C* to a majority gate are stored as resistance $R_{A},R_{B},R_{C}$ which are either in HRS or LRS in cell locations (1,1), (2,1) and (3,1). To compute majority, $WL_{1}$, $WL_{2}$ and $WL_{3}$ are activated simultaneously and a voltage $V_{R}$/3 is applied across the 1T–1R cells in col.1 ($BL_{1}$–$SL_{1}$). Since one terminal of all RRAMs in col.1 is connected to $BL_{1}$ and the source terminal of all transistors in col.1 is connected to $SL_{1}$, the resistances *A*, *B* and *C* will be in parallel (neglecting the interconnect resistance of BL/SL) resulting in a current, $I_{MAJ}$ = $\frac{V_{R}/3}{R_{A}\left|\right|R_{B}\left|\right|R_{C}}$. In other words, $I_{MAJ}$ = $I_{A}$+$I_{B}$+$I_{C}$, where $I_{A}$, $I_{B}$, $I_{C}$ are the currents through individuals cells *A*, *B* and *C*. When two out of the three cells are in LRS, we have a higher current compared to the case when two out of the three cells are in HRS. A current mode SA (Section 2.2) distinguishes between these currents to convert $I_{MAJ}$ to a voltage, which results in MAJ(A,B,C). The output of the majority gate (i.e., the SA’s output) can be written into the array in the next cycle for further processing.

To validate the proposed gate, the 1T–1R cell from IHP (Innovations for High Performance Microelectronics– Leibniz-Institut für innovative Mikroelektronik, Germany) is considered. The 1T–1R is constituted by NMOS transistor manufactured in IHP’s 250 nm CMOS technology, whose drain is connected in series to the RRAM. The RRAM is a $TiN/Hf_{1-x}Al_{x}O_{y}/Ti/TiN$ stack integrated on the metal line 2 of the CMOS process. In an earlier work [13], IHP’s 1T–1R cells were modeled by fitting Stanford-PKU RRAM model to the measurement data. The cells have a median LRS and HRS of 6.6 KΩ and 66.6 KΩ, respectively. To read from the cells, a voltage of 1.4 V is applied to the gate (*WL*) and a read voltage of 0.2 V is applied across the cell (BL-SL) resulting in $I_{HRS}$ and $I_{LRS}$ of 3 μA and 30 μA, respectively. To use the same SA to read single cell and to compute majority (three cells are read), we employ two different read voltages (the current mode SA of Section 2.2 has to use the same $I_{REF}$ to read, as well as to compute majority). We use a $V_{R}$ of 0.3 V to read a single cell, resulting in $I_{HRS}$ and $I_{LRS}$ of 4.5 μA and 45 μA, respectively. This does not affect the resistive states since IHP’s cells are SET and RESET at 0.9 V and −1.1 V, respectively. To compute majority, we employ a voltage of $V_{R}$/3 which should result in a current, $I_{MAJ}$ of $\frac{V_{R}/3}{R_{A}\left|\right|R_{B}\left|\right|R_{C}}$ (Table 1). We simulated a 64 × 64 1T–1R array (including interconnect parasitics) and the resulting current is denoted $I_{MAJ}^{sim}$ in Table 1. Due to the parasitic resistance of the bitline, the effective voltage across the cell is reduced, resulting in a small mismatch between $I_{MAJ}$ (calculated) and $I_{MAJ}^{sim}$ (simulated).

### 2.2. Sensing Methodology

**Figure 5:**
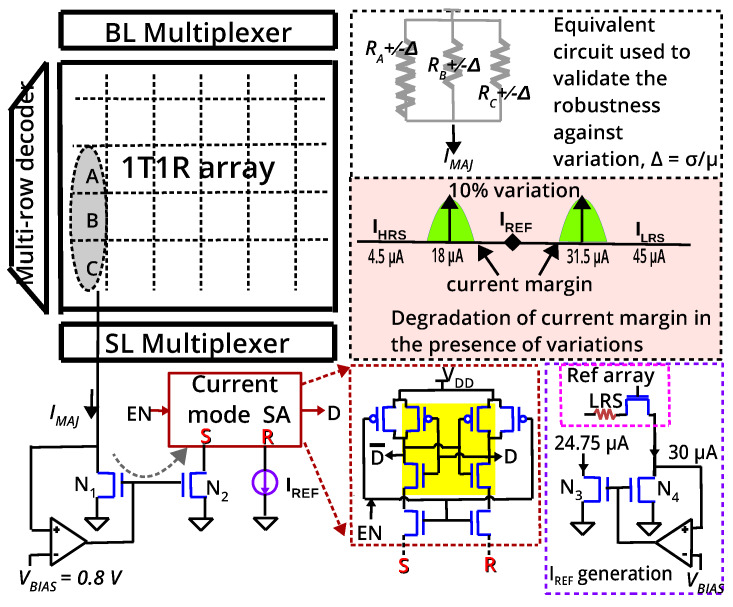
Current in the sense path (‘S’) and reference path (‘R’) are compared. ‘*D*’ and ‘$\bar{D}$’ are precharged to $V_{DD}$ when EN is low. When EN goes high, one of them discharges at a faster rate, which is reinforced by the positive feedback formed by cross-coupled inverters (shaded yellow) [14,15].

The methodology to reliably translate $I_{MAJ}$ into a CMOS compatible voltage is the crucial aspect of the proposed majority gate implementation. We used the sensing method presented by Woorham Bae et al. [14,15] which is based on StrongARM latch to sense current difference of the order of a few μA (Figure 5). The current $I_{MAJ}$ from the 1T–1R array is mirrored by $N_{1}$-$N_{2}$ pair and compared with $I_{REF}$ in a current mode SA. The op-amp biases the drain of transistor $N_{1}$ at a constant voltage, $V_{BIAS}$ to ensure that $N_{1}$ is in saturation (feedback bias [15]). To read from a cell, $V_{BIAS}$ of 0.8 V was used and 1.1 V was applied at BL, resulting in an effective voltage of 0.3 V (the SL is held at 0.8 V by the op-amp, Figure 5). Consequently, the SA has to distinguish between 4.5 μA (HRS) and 45 μA (LRS). To compute majority, $WL_{1-3}$ were activated and 0.9 V was applied at the BL and hence, the effective voltage across the cells is only 0.1 V ($V_{R}/3$). In this case, the SA has to distinguish between currents ≤18 μA and ≥31.5 μA (highlighted in Table 1). We chose $I_{REF}$ to be 24.75 μA to maximize the sensing margin (Reference current $I_{REF}$ is usually generated from a reference array where some dummy cells are programmed to LRS; a voltage of 0.2 V across the cell produces 30 μA which can be scaled to 24.75 μA by the current mirror, Figure 5). Therefore, the majority gate can be implemented in a regular 1T–1R array without any change in the sensing circuitry.

**Figure 6:**
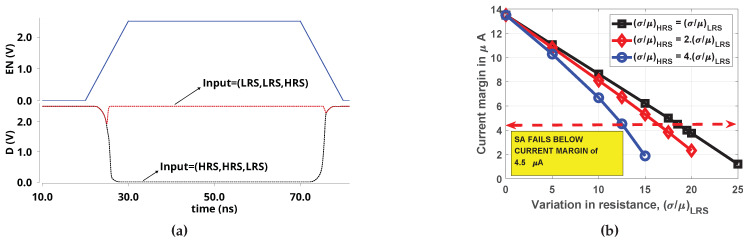
(**a**) A sample output of the Sense Amplifier (SA) for a typical case of sensing with 10% variation in LRS and 40% variation in HRS. EN is the Sense amplifier’s enable signal and, as soon as it goes high, the SA output node (D) is discharged to 0 V (HRS,HRS,LRS) or remains high (LRS,LRS,HRS) (**b**) Effect of variations in IHP’s cells on current margin.

RRAMs exhibit inevitable variability in HRS/LRS states and the SA must be able to distinguish between weak minority (two HRS and one LRS) and weak majority (two LRS and one HRS), in the presence of variations (usually quantified as relative spread, σ/μ, where σ is the standard deviation and μ is the mean resistance of the state). Furthermore, many studies have revealed that variability is larger at HRS than at LRS [16,17,18,19] due to stochastic nature of the filament rupture (few studies have compared the actual variation in HRS and LRS, e.g., in a $HfO_{x}$ device, the reported variation is 16% at LRS and 36% at HRS. For $TiO_{x}$ device, it is 14% at LRS and 26% at HRS [20]). Since the RRAM is not switched while computing majority (the gate ‘reads’ majority and it is not affected by variations in $V_{SET}/V_{RESET}$ voltages), the equivalent circuit depicted in Figure 5 was used to study robustness against variations. The current margin is the difference in the current drawn (between weak minority and weak majority) from the equivalent circuit, which has to be sensed correctly by the SA, i.e., in the absence of variations, the current margin is 31.5 μA − 18 μA = 13.5 μA. In Figure 6, we plot the degradation in current margin with respect to variation, σ/μ, for the worst case, that is, σ/μ of 10 signifies LRS of cell at a particular location may be μ + 10% at the present cycle and may be μ − 10% at the same location after a subsequent ‘write’ operation (cycle-to-cycle). Alternatively, LRS of a cell in column 1 may be μ + 10% and LRS of cell in column 64 may be μ − 10% (device-to-device). In both cases, a SA with the same $I_{REF}$ must be able to ‘read’ majority correctly. For IHP’s RRAM, the gate can tolerate upto 18.5% variations (when the quantum of variation in HRS and LRS are same) and upto 12.5% variations in LRS (when the variation in HRS is four times the variation in LRS). Interestingly, increased variations in HRS does not proportionately degrade the current margin since $I_{MAJ}$ is dominated by LRS (Figure 6b).

### 2.3. Adapting the Majority Gate to Other RRAM Technologies

To study the generality of our approach to other RRAMs ($TaO_{x}$,$SiO_{x}$ etc.), we considered a $Ti/SiO_{2}/C$ device recently presented in Reference [17]. The device has a mean LRS and HRS of 20 KΩ and 100 MΩ, respectively (resistance window = 5000). When the cell is read with 0.1 V, $I_{LRS}$ and $I_{HRS}$ will be 5 μA and 1 nA. Consequently, one can verify that $I_{MAJ}$ will be 5.002 μA and 10.001 μA for weak minority and majority, respectively and, the current margin is reduced to only 5 μA. To increase the current margin, we exploited the current mirror formed by transistors $N_{1}$–$N_{2}$ to multiply $I_{MAJ}$ by three, and fed it to the current mode SA (i.e., ${\left(\frac{W}{L}\right)}_{N2}$ was made $3\times {\left(\frac{W}{L}\right)}_{N1}$ in Figure 5). Now, the SA has to distinguish between 15 μA and 30 μA and $I_{REF}$ was chosen to be 22.5 μA. For this device, correct sensing was verified with upto 24% variations in LRS and 100% variation at HRS (for this device, even a 400% variation in HRS will not affect sensing because the corresponding effect in $I_{MAJ}$ will be in nA). Therefore, for RRAMs with higher resistance window, the majority gate is completely immune to variations in HRS.

## 3. Framework to Compute in 1T–1R Array

### 3.1. Multi-Row Decoder Design

**Figure 7:**
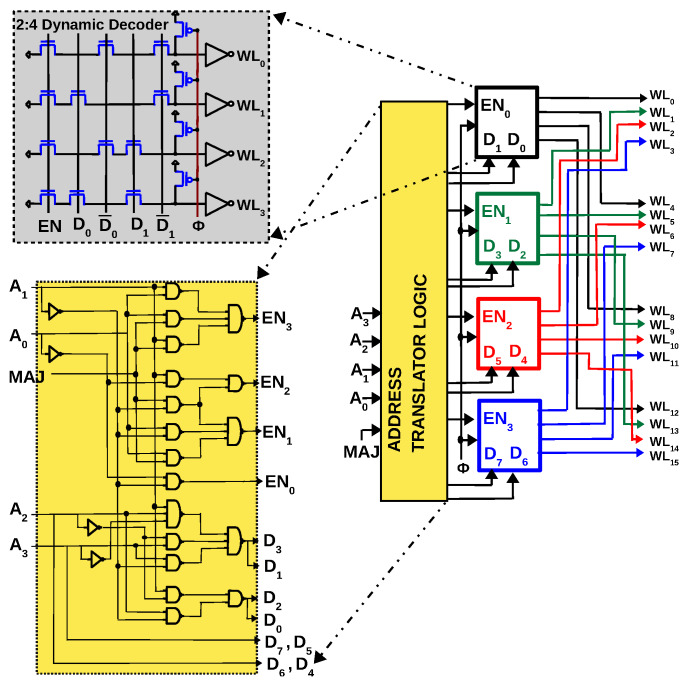
Multi-row decoder is implemented by interleaving multiple decoders and using an additional control signal called MAJ. When MAJ is logic ‘0’, $WL_{i}$ corresponding to row address $A_{3}A_{2}A_{1}A_{0}$ is selected. When control signal MAJ is logic ‘1’, $WL_{i},WL_{i+1},WL_{i+2}$ are selected.

Since the same SA is used to read one bit as well as compute majority of three bits, the only significant change in the peripheral circuit of the 1T–1R array will be the row decoder. A conventional 1T–1R memory can select one row at a time, while the proposed gate needs three rows to be selected simultaneously. As depicted in Figure 7, a 4:16 multi-row decoder can be designed by interleaving four 2:4 dynamic NAND decoders (a dynamic decoder uses a precharge signal ϕ, which when low, all WL are driven to ‘0’. When ϕ goes high, $WL_{i}$ corresponding to $D_{1}D_{0}$ goes high provided EN is ‘1’). Since single-row decoding must co-exist with multi-row decoding, an address translator circuit is used to switch between the two modes using MAJ as a control signal. A single row can be selected (e.g., $WL_{5}$) by enabling the green decoder ($EN_{1}$ = 1) and setting ($D_{3}D_{2}$) to (01). All other decoders are disabled ($EN_{0}$,$EN_{2}$,$EN_{3}$ = 0) and hence no other WL is selected. During majority operation, the same address ($A_{3}A_{2}A_{1}A_{0}$ = 0101) will not only select $WL_{5}$, but also $WL_{6}$ and $WL_{7}$. This is achieved by enabling three decoders ($EN_{1}$,$EN_{2}$,$EN_{3}$ = 1) and setting ($D_{7}D_{6}D_{5}D_{4}D_{3}D_{2}$) to (010101) while $EN_{0}$ is set to 0. The ‘Address translator’ inputs MAJ and $A_{3}A_{2}A_{1}A_{0}$, and generates $D_{7}D_{6}D_{5}D_{4}D_{3}D_{2}D_{1}D_{0}$ and $EN_{3}EN_{2}EN_{1}EN_{0}$ to achieve this desired functionality.

### 3.2. Functional Completeness and One-Bit Full Adder

**Figure 8:**
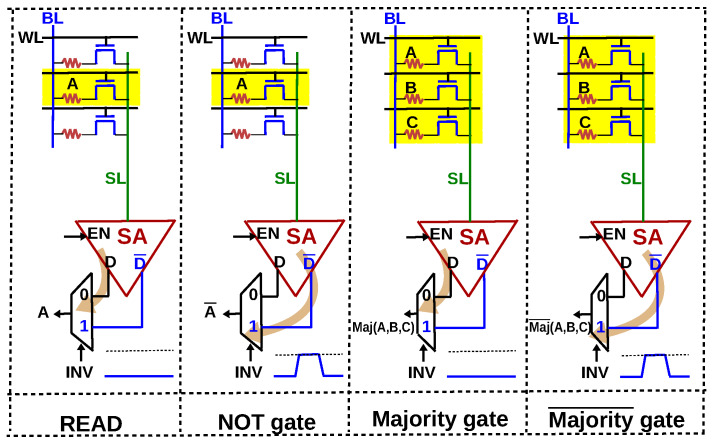
With an additional 2:1 Mux at the output of the SA, a NOT gate can be implemented by using the control signal INV. Similarly, majority and its complement, $\bar{majority}$ can be implemented. All logic operations are essentially READ operations.

**Figure 9:**
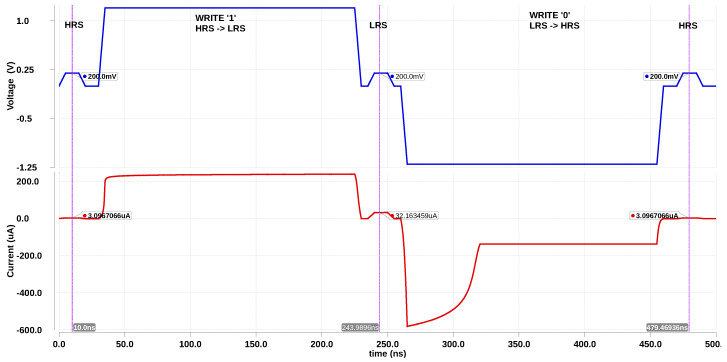
Voltage across the RRAM (BL–SL) and the current during WRITE operations. The state of the RRAM cell is detected by a small voltage applied across the cell and measuring the current through it.

The current-mode SA used in Figure 5 outputs data (*D*) and its inverted value ($\bar{D}$). We exploit this capability of the SA to implement all the basic operations needed for implementing Boolean logic, as depicted in Figure 8. If control signal INV is high, it reads the inverted value of a particular bit, resulting in a NOT gate. Similarly, we can implement majority gate and its inversion during READ by using the control signal INV. In a conventional memory, the memory controller is a circuit which orchestrates READ and WRITE operations. This is achieved by translating the READ and WRITE commands to appropriate control signals, which are in turn applied to the peripheral circuitry of the array. Since logic operations are implemented as READ operations, computing capability can be incorporated into the memory controller by including MAJ and INV as two additional control signals. Table 2 lists the memory operations (READ,WRITE) and logic operations (majority, NOT, $\bar{majority}$), and the associated control signals activated to implement them. During logic operations and READ, the SA is enabled (EN is high) and during WRITE operation, it is disabled (EN is low). WRITE ‘1’ is accomplished by applying a voltage pulse ($V_{SET}$) to the BL while SL is grounded. The RRAM transitions from a HRS to a LRS as shown in Figure 9. WRITE ‘0’ is accomplished by applying a voltage pulse ($V_{RESET}$) to the SL while BL is grounded. The RRAM transitions from a LRS to a HRS as shown in Figure 9.

**Figure 10:**
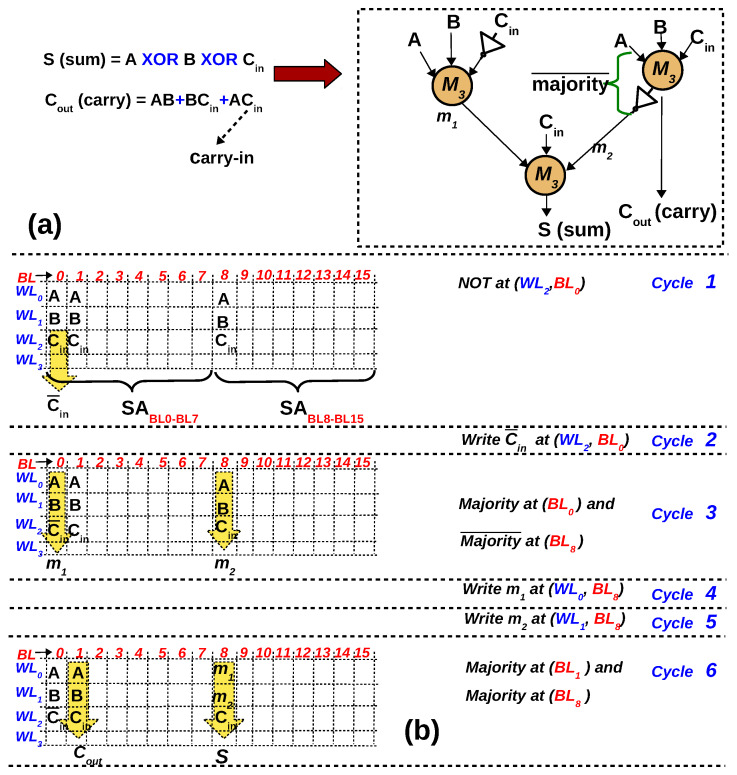
(**a**) 1-bit full adder in majority logic, where $M_{3}$ represents 3-input majority gate [21] (**b**) Mapping of 1-bit adder to 1T–1R array.

It must be noted majority and NOT together form a functionally complete logic, i.e., any Boolean logic can be expressed in terms of majority and NOT gates [5]. Figure 10a is a 1-bit full adder expressed in terms of majority and NOT gates [21]. Any Boolean logic can be synthesised in terms of majority and NOT gates using logic synthesis/optimization methods [22]. A 1-bit full adder can be executed in the 1T–1R array in six cycles, as elaborated in Figure 10b. It is assumed that the inputs to the full adder ($A,B,C_{in}$) are arranged in the memory array as depicted in the 4 × 16 array in Figure 10b. Further, it is assumed that 8 BLs share a SA, similar to a 1T–1R chip fabricated in Reference [23], which uses a similar current-mode SA. The mapping from the circuit in Figure 10a to 1T–1R array is straightforward once the inputs to the gates are aligned in a column. Based on the input data dependencies of the gates, two gates can be executed in parallel in step 3 and 6 and the ‘Sum’ and ‘Carry’ bits will be available in the SA after 6 memory cycles. In cycle 3, $\bar{majority}\left(A,B,C_{in}\right)$ is needed at $BL_{8}$ because executing majority followed by NOT gate will increase the latency. When mapping large circuits, scheduling algorithms such as the ones used in high-level synthesis can be used to distribute the gates uniformly between levels such that gates at each level can be executed in parallel, thereby reducing latency.

### 3.3. Comparison with Other In-Memory Adders

Usually, the speed of memristive logic families are compared in terms of steps or memory cycles and not time (frequency) [12,24,25] This is because, the switching times (HRS → LRS) of these devices vary from lab to lab (switching time depends on various properties of the device like switching oxide, electrodes, SET/RESET voltage used etc.). Unlike CMOS technology, there is no standardized technology for RRAM. Reported RRAM devices have switching times varying from a few *n*s to μ seconds. So latency can be used to compare the speed of different in-memory computing approaches. A comparison of the latency of ‘in-memory’ 1-bit full adders using different logic primitives, is presented in Table 3. As summarized in Table 3, the number of steps to compute in a RRAM array, reduces significantly from IMPLY to NAND/NOR logic primitive, and, further from NAND/NOR to majority, proving the strength of majority as a logic primitive. The energy for one-bit addition can be calculated by summing the energy for different logic operations. The energy for READ operation was calculated by integrating the current (in the current–mode sense amplifier) and found to be 8.44 pJ. The energy for WRITE operation was calculated by integrating the current through the RRAM cell (Figure 9) and found to be 46 pJ. Therefore the energy for one-bit addition is 180 pJ (five READ and three WRITE operations, Figure 10b). In Table 3, the energy for different one-bit adders is not included since the energy of other adders is either not reported or reported for another RRAM technology. As stated earlier, it would be unfair to compare the energy across different RRAM technologies since the switching energy (HRS↔LRS) depends on HRS and LRS values and also the switching time. However, latency can be an approximate measure of energy consumption (energy ∝ latency) and accurate energy comparison can be performed by simulating all the adders with the same memristive device.

## 4. Conclusions

A majority gate can be implemented in a 1T–1R array without necessitating any change in the sensing circuitry. For RRAMs with higher resistance window, the gate is completely immune to HRS variations and is affected only by LRS variations, which is dictated by the thickness of the filament and hence well controlled by the compliance current. Computing capability can be augmented to the regular memory with a minor modification to the row-decoder of the array. Both majority gate and NOT gate can be implemented as READ operations. In this manner, a one-bit full adder can be implemented in 6 memory cycles, where each cycle is a memory READ/WRITE operation. A 40% reduction in latency is achieved for the realisation of a one-bit full adder, when compared to NAND/NOR logic primitive. In future, the author wants to investigate how majority logic can reduce the latency of larger in-memory circuits. Although only RRAM was analysed in this article, the presented computing method can be adopted for any memristive device fabricated in a 1T-1R configuration, with a resistance window ≥ 10 (the RRAM device studied in this work has a resistance window of 10, for devices with lower resistance window, sensing majority becomes a challenge). Therefore, this contribution has a wide application in the in-memory computing landscape intensively researched in recent years.

## Figures and Tables

**Table 1:** Majority logic can be implemented by accurately sensing $I_{MAJ}$: Logic ‘0’ is HRS and logic ‘1’ is LRS. The sense amplifier has to distinguish between the rows shaded grey and those that are not.

*A*	*B*	*C*	MAJ(A,B,C)	$I_{\mathrm{MAJ}}$	$I_{\mathrm{MAJ}}^{\mathrm{sim}}$
0	0	0	**0**	4.5 μA	4.27 μA
0	0	1	**0**	18 μA	17.54 μA
0	1	0	**0**	18 μA	17.54 μA
0	1	1	**1**	31.5 μA	30.75 μA
1	0	0	**0**	18 μA	17.54 μA
1	0	1	**1**	31.5 μA	30.75 μA
1	1	0	**1**	31.5 μA	30.75 μA
1	1	1	**1**	45 μA	44.03 μA

**Table 2:** Control signals activated to implement memory and logic operations ($V_{R}$ is read voltage).

Memory/Logic Operation	WL	BL	SL	EN(Sense Amplifier)	INV	MAJ
READ	single row activated	$V_{R}$	connected to SA	1	0	0
NOT	single row activated	$V_{R}$	connected to SA	1	1	0
Majority	three rows activated	$V_{R}$/3	connected to SA	1	0	1
$\bar{Majority}$	three rows activated	$V_{R}$/3	connected to SA	1	1	1
WRITE ‘1’	single row activated	$V_{SET}$	grounded	0	0	0
WRITE ‘0’	single row activated	grounded	$V_{RESET}$	0	0	0

**Table 3:** Latency of one-bit full adders implemented in RRAM array.

Primitive	Structure	Latency	Area	Ref.
IMPLY	1R	27 steps	4 × 2	[26]
IMPLY (semi-parallel)	1T–1R	17 steps	5	[24]
ORNOR	1T–1R	17 steps	12	[25]
NOR	1S–1R	10 steps	12 × 4	[6]
NAND	1S–1R	10 steps	1 × 9	[27]
Majority+NOT	1T–1R	6 steps	3 × 9	This work
